# Mapping saline water intrusion into the coastal aquifer with geophysical and geochemical techniques: the University of Lagos campus case (Nigeria)

**DOI:** 10.1186/2193-1801-2-433

**Published:** 2013-09-04

**Authors:** Elijah A Ayolabi, Adetayo F Folorunso, Abiodun M Odukoya, Adelere E Adeniran

**Affiliations:** Department of Geosciences, University of Lagos, Lagos, Nigeria; College of Marine Geosciences, Ocean University of China, Qingdao, 266003 China; Works and Physical Planning Department, University of Lagos, Lagos, Nigeria

**Keywords:** Saltwater intrusion, ERT, Coastal aquifer, Geochemical study, Pollution index, Delineation

## Abstract

Saltwater intrusion into the coastal aquifer, a phenomenon brought by the flow of seawater into freshwater aquifers originally caused by groundwater extraction near the coast, has long been recognised as a major concern around the world. In this study, we employed geophysical and geochemical techniques to map and provide evidences that the coastal aquifers in the study area have been intruded by saltwater from the adjacent Lagos lagoon. The resistivity data were acquired with an electrode spacing (a) that vary between 1.6 to 8 m, and expansion factor n of 30. The depth inverted models obtained from inversion of the fifteen resistivity data obtained in the area revealed significant impact of the lagoon water on the aquifers indicated as low resistivity usually below 7 Ωm. A combination of four different electrode arrays – Schlumberger, Wenner, Dipole-dipole and pole–dipole, with at least three deployed at each site ( except for three traverses – traverses 13, 14 and 15), yield better horizontal and vertical resolution, having depth range of 36–226 m with 1.6–8 m electrode spacing used. The delineated geoelectric layers were juxtaposed with logs from both boreholes located within the campus. Evidence from geochemical study of borehole and the lagoon water samples corroborated the ERT result. Progressive decrease in total dissolved solute (TDS) and electrical conductivity (EC) from the lagoon to the coastal aquifer buttresses gradual encroachment of the inland aquifers by the intruding lagoon water. In addition, similar trend was observed in heavy metal distribution Pollution Index (PI) plot suggesting possible underground flow of water from the lagoon to the aquifers. From this study, we deduced that excessive groundwater extraction and possibly the reduction of groundwater gradients which allows saline-water to displace fresh water in the aquifer of the investigated area are responsible for the saline water intrusion observed.

## Introduction

Saltwater intrusion into the coastal aquifer has long been recognised as a major concern around the world. The intrusion occurs as a result of the induced flow of seawater into freshwater aquifers originally caused by groundwater development around the coast (Al Barwani and Helmi, 2006). The induced gradients usually cause migration of salt water from the sea toward a borehole where groundwater is being pumped from aquifers that are in hydraulic connection with the sea (Abdalla et al., 2010). This will definitely make the freshwater in the well unusable and thus hamper the development of potable water for municipal use. Almost two thirds of the world’s population are said to be living within 400 km of the ocean shoreline and just over half live within 200 km, an area only taking up 10% of the earth’s surface (Hinrichsen, 2007). These coastal dwellers depend largely on groundwater as main source of fresh water for daily survival, industrial and agricultural purposes; a development that could lead to excessive groundwater extraction.

Basically, fresh water is less dense than salt water and so it floats on top. Therefore, saline water is found below fresh water discharge from higher altitude in coastal areas. Also, the boundary between salt water and fresh water is not distinct; the zone of dispersion, transition zone, or salt-water interface is brackish with salt water and fresh water mixing. It then means that salinity will increase with depth where both fresh water and saline water occur. As such, the increase in salinity will produce consequent decrease in electrical resistivity of water, and thus resistivity varies with depth within groundwater well in coastal aquifer. These variations could then be mapped by methods capable of detecting differences in salinity. Thus, we employed electrical resistivity, geochemical and physical measurements of well water and seawater samples in this work.

Electrical methods have been used to solve problems related to environmental studies, especially relating to contamination of subsurface soil and groundwater and aquifer vulnerability (Loke, 2004; Sorensen *et al*., 2005; Atakpo and Ayolabi, 2009; Gemail *et al*., 2011, Vaudelet et al., 2011). Vulnerability of groundwater aquifer is defined as the susceptibility of a given aquifer to pollution (Younger, 2007). Many authors see aquifer vulnerability from the perspective of anthropogenic input into groundwater, especially from the surface, without recourse to underground movement of materials (soil, minerals and water) – internal erosion devoid of human influence. Sjödahl, et al. (2008) noted that the knowledge of the temporal development of internal erosion is limited. In coastal regions, many factors contribute to such movement which include, but not limited to, salt water intrusion owing to massive withdrawal from the adjoining inland groundwater aquifers.

### Geology setting and site description

**Figure 1:**
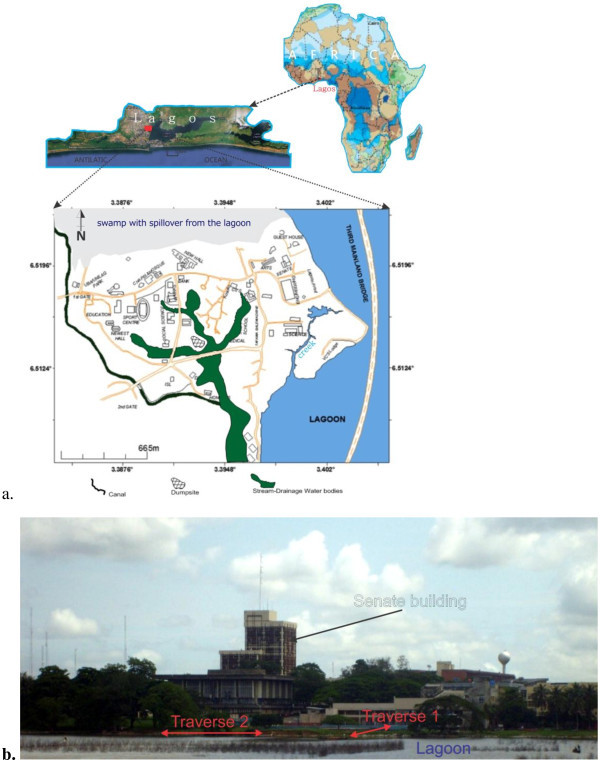
**a) The geography of the University of Lagos campus b) The University building showing the Lagoon and some ERT lines.**

University of Lagos is located within Lagos, Southwestern Nigeria. Geographically, the campus is surrounded by water bodies; lagoon and creek, and has large tract of land (Figure 1a and b). It is generally low-lying and characterized by torrential rainfall and a shallow water table (as shallow as 0.5 m around the coast). The geology of the area is characterized by two bands of sand separated by silty mud. It lies within the Dahomey Basin. The age of the Sedimentary formations of Lagos area could be described as ranging from the Cretaceous through Tertiary to Quaternary sediments. Abeokuta group are Cretaceous sediments while Ilaro, Oshosun, Akinbo and Ewekoro Formations are Tertiary (Obaje, 2009). Quaternary sediments are alluvial deposits, covering most part of the Lagos Coastal areas and river valleys. For details lithographic succession of Dahomey Basin of which Lagos is an integral part, readers can refer to Okosun (1990) and Obaje (2009).

**Figure 2:**
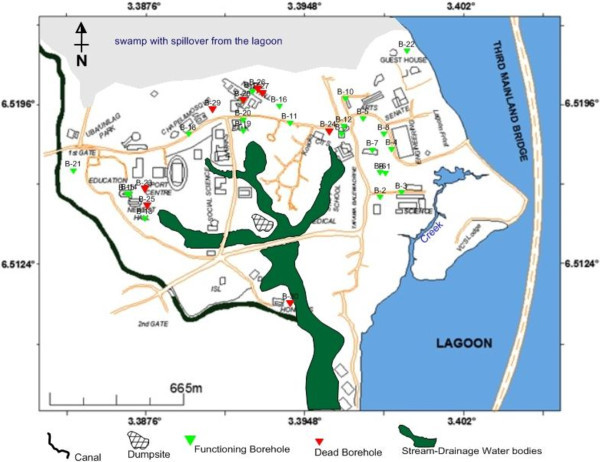
**Location map of the known boreholes drilled to date within the campus.** Over 70% of these wells are still functioning and extracting groundwater at the time of this survey.

The University with population of students and staff totalling about fifty thousand depends on its own boreholes for the water supply; hence, this has led to disproportionate sinking of boreholes to meet the water need of the campus community. Over thirty (30) water boreholes were located within the few acres of land the Institution occupies, with at least twenty (20) of the boreholes functioning as at the time of this investigation (Figure 2). They are capable of generating massive groundwater extraction, which poses a great deal for the recharge of aquifer in this area. We applied methods which would be able to image the subsurface and delineate saline-fresh water contact and possible flow path of any intruding saltwater.

## Data acquisition

Methodologies adopted in the study were a combination of geophysical survey and physico-chemical measurements to evaluate aquifer vulnerability and extent of saline water encroachment within the University campus. The combination is aimed to enhance details of the subsurface – both soil and water, with the understanding that whatever affects the soil has consequent effects on the water.

### Geophysical data

#### Electrical resistivity tomography

Electrical resistivity tomography (ERT) survey was carried out using AGI SuperSting R8/IP8 Earth Resistivity meter, an 8 channel Memory Resistivity and IP Meter with inbuilt processor for 64 and 84 multi-electrodes system. For better horizontal and vertical resolution, four types of electrode array were deployed for the investigation – Schlumberger, Wenner, Dipole-dipole and pole – dipole with three in operation at each site, except line 1, 3, 13, 14 and 15 due to some logistics. There were variations in the minimum electrode spacing ‘a’ used (from 1.6-8.0 m) owing to constraint in space since the entire campus is built up. Notwithstanding, a fairly deeper depth were probed in the traverses, having used expansion factor n = 30.

The choice of arrays for the survey was based on our desire to have good sensitivity to vertical and horizontal changes in the subsurface resistivity at both shallow and deeper depths, at the same time probe into the deeper depth and to have good vertical and horizontal data coverage under each ERT line. Despite possible noise from other activities on the campus, we still want the best result. We therefore chose the arrays to meet these demands. Wenner array is known in its ability to resolve vertical changes (horizontal structures), has a moderate depth of investigation though poor at horizontal coverage upon increasing the electrode spacing (Loke 2000). To make up for this, we employed Dipole-Dipole (Douple-Dipole) which is good in mapping vertical structures, as well has a better horizontal data coverage than the Wenner (Loke, 2004). Pole-Dipole gave the highest depth (226 m) to satisfy our intention for deeper depth. We also chose Schlumberger for 2-D survey array with overlapping data levels because of its good horizontal and vertical resolution.

**Figure 3:**
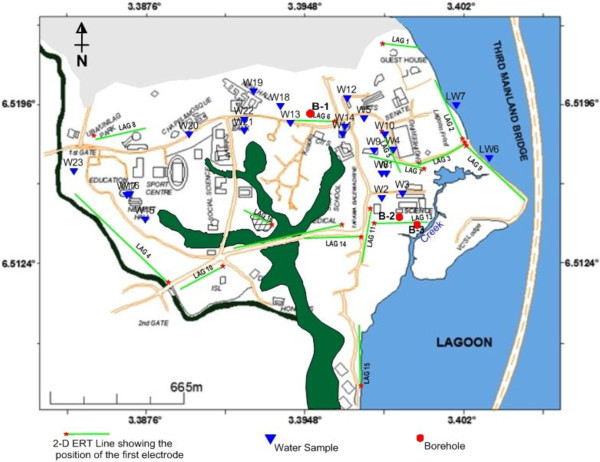
**Locations of ERT survey lines, borehole and lagoon water samples and boreholes logs relative to building structures within the campus.**

In all, fifteen (15) 2-D ERT profiles (Figure 3) were measured covering the whole coastal area. One of the latest softwares for the inversion of 2-D data, the Earth Imager, was used to invert the apparent resistivity profiles to obtain a 2-D image of the subsurface resistivity distributions. The ERT profiles were inverted using an iterative smoothness constrained least-squares inversion algorithm otherwise known as “OCCAM inversion” after deGroot-Hedlin and Constable (1990) and Loke and Barker (1996). These inversion routines involve a cell-based inversion technique; it subdivides the subsurface into a number of rectangular cells in which resitivities are varied to obtain the best fit with the observed data (Loke, 2004). The differences between the observed and calculated data are minimized to obtain an acceptable agreement (Loke and Barker, 1996). A measure of this difference is given by the root-mean-square error (RMS%). However, smoothness constrained models do not allow for large and unrealistic variations in the output models, as its name suggests. Notwithstanding this, a reasonable value of RMS (not shown) was maintained for all the profiles.

Efforts were made to distribute some profiles from the lagoon progressively into the main land (the campus), where possible, to monitor the progression of saline water intrusion into the aquifer within the study area. In addition, some profiles were also positioned close to existing boreholes where possible to constraint the results. For this purpose only one existing and two geotechnical boreholes drilled during the course of our survey were used for correlation.

### Geochemical data

Water samples from twenty one boreholes and two lagoon water were collected at various locations within University of Lagos (Figure 3). Eight boreholes were not functioning at the time of this study. Several sensitive parameters of water such as total dissolved solids (TDS), electrical conductivity (EC), temperature and pH were determined in situ using digital meters (e.g. water treatment works (WTW)-conductivity meter model L/92 and WTW- pH meter model pH/91). The meter was calibrated with pH solutions 4 and 7. Water samples of approximately 125 mL were collected for multi-element analysis; pressure filtered through 0.2 mm Nuclepore membranes and 3 mL analytical grade HNO_3_ was added to bring the water acid solution to approximate pH of 2.

The analysis of trace elements and cations in water were carried out using inductively coupled plasma optical emission spectrometry (ICP-OES) while unacidified water samples were analyzed for anions concentrations using the DIONEX DX-120 ion chromatography techniques. All the analyses were carried out at the ACME laboratory, Ontario Canada. The samples were analyzed for 73 constituents and physical properties. To check the accuracy, activation laboratory employed two internal standards (each run twice) and found that the errors were consistently minimal. Results were further compared with recommended standards and pollution index was calculated to determine the water quality. Analytical results for significant elements were compared with United State Environmental Protection standards (USEPA). The standards include Maximum Contaminant Levels (MCLs), Secondary Maximum Contaminant Levels (SMCLs) established by the USEPA (2002,2004 and 2009). MCLs are enforceable standards that specify the highest level of a contaminant that is allowed in drinking water. Details of hydrogeochemistry of the study area evidencing and detailing chemistry and spatial evolution are presented in another paper currently in preparation.

## Result and interpretation

### 2-D Electrical Resistivity Tomography (ERT)

**Figure 4:**
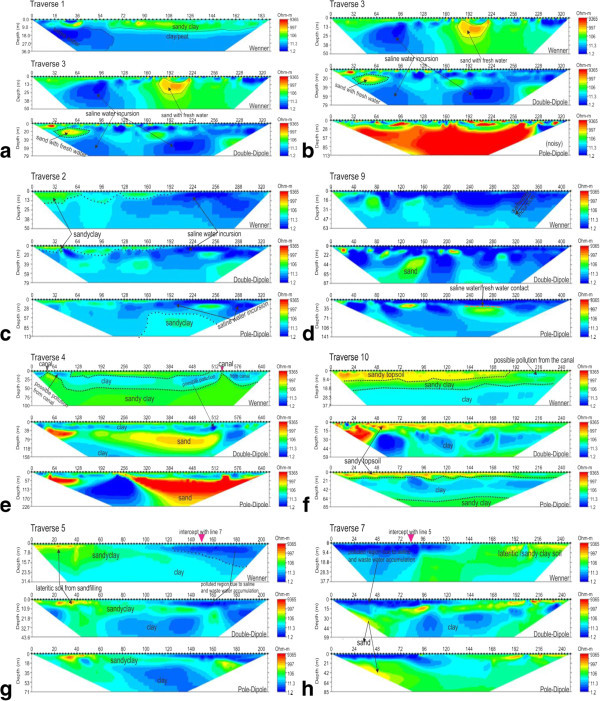
**Earth imager inverted resistivity-depth models for the ERT lines for traverses 1 to 5, 7, 9 and 10 (a to h).** Note the high conductivity under traverses 1, 2, 3 and 9 **(a, c, d, b** respectively**)** at highest proximity to the lagoon; also note the similarity in inverted resistivity-depth models at the intercepting section of traverses 5 and 7 **(g and h)**.

**Figure 5:**
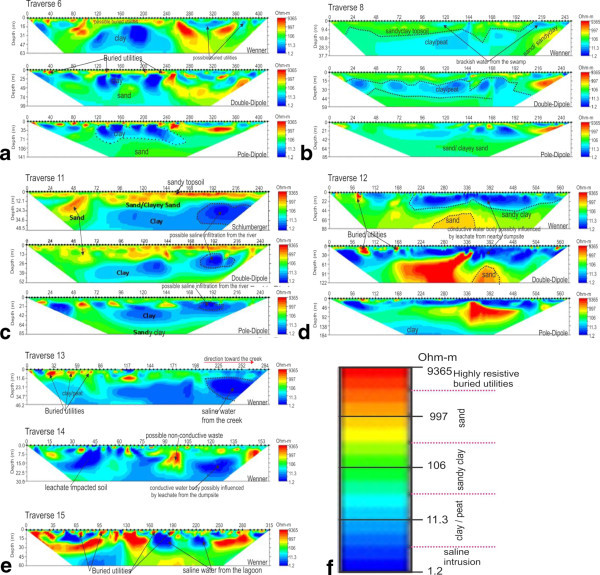
**Earth imager inverted resistivity-depth models for the ERT lines for traverses 6, 8, and 11 to 15 (a to e) and (f) interpretations of geoelectric layers.** Note that F is a logarithm scale.

Fifteen (15) 2-D Electrical Resistivity tomography profiles measured within the available space have lateral extent ranging from 135 to 664 m and vertical extent of 45 to 226 m. The data were processed and inversion to obtain the 2-D ERT models displayed in Figures 4 and 5. The 2-D ERT sections clearly indicate variation in subsurface conductivity/resistivity which reflects different lithology and fluid content as marked in Figure 5f. The subsurface layers consist of top soil with varying materials of low to high resistivity value, clay, peat, sandy clay/clayey sand and sand. The sandy clay/clayey sand and sand layers constitute the aquifer unit with varying ground water yield due to its varying porosity and permeability. Some of the aquifer units were found to have been impacted by saline/brackish water due to saltwater incursion from the Lagoon and are characterized by very low resistivity value. Other sources of pollution observed from our results include saltwater infiltration from the creek, waste water accumulation and percolation through unlined drainage/canal within the university, waste water from adjoining canal, infiltration from polluted stream. Leachate from the dumpsite within the campus may become another major source of pollution to the groundwater with time.

The 2-D ERT sections along traverses 3 and 1 were taken perpendicular to the Lagoon to monitor the progressive seawater incursion to the neighbouring coastal aquifers. Resistivity distributions indicate salt water incursion from the lagoon extending to a depth of about 35 m (Traverse 1) and 45 m (Traverse 3) beneath the surface marked with very low resistivity value ranging from 0.3–10.0Ωm. Ground surface under Traverse 1 from surface positions 60 m to 190 m was a sand-filled land reclaimed from lagoon prior to the erection of the University Guess House building. This was well noted during the field survey. This possibly accounts for the thick high resistive material display above the saline water intrusion zone in the resistivity structure (Figure 4a). Pollution observed within the subsurface along this traverse was probably due to combination of saltwater incursion from the lagoon and waste water percolation into the subsurface along the drainage/waste water channel. But near surface high resistivity structure at isolated points (Figure 4b) along the traverse was due to the existence of buried drainage/bridge/high resistivity exotic materials.

2-D ERT sections for Traverses 2 and 9 were taken parallel to the lagoon probing a vertical depth of 50–113 m and 63–141 m respectively. The subsurface within this area is composed of predominantly low resistivity (0.11–5.0 Ωm) structure reflective of saline water incursion in to the aquifer. Figure 4c (Traverse 2) show that the entire traverse has been impacted by the lagoon water incursion to a depth of 40–85 m beneath the surface while Figure 4D (Traverse 9) clearly indicates Lagoon water invasion of the fresh water aquifer to a depth of 0–42 m; both at higher depths due to high proximity to the lagoon. This clearly shows that the first and the second aquifer have been polluted by the saltwater incursion from the lagoon.

Traverses 4 was run parallel to and Traverse 10 perpendicular to a major canal that demarcates southern and western sides of the campus from surrounding communities. 2-D ERT model along Traverse 4 (Figure 4e) shows possible brackish water pollution from the carnal (very low resistivity, 1–20 Ωm) beneath electrode positions 0–88 m and 424–640 m at a near surface depth of 0–25 m. At surface positions 424–640 m, the pollution falls within the first aquifer unit possibly prevented from further percolation by the underlying thin clayey layer (less than 50 Ωm). Similarly, low resistivity value of 10–20 Ωm (Traverse 10) was noticed within near surface depth of 0-9 m at a lateral distance of 195–225 m towards the Canal (Figure 4f) suggestive of the same possible brackish water invasion of this layer. The underlain sand/clayey sand is the aquifer unit and is prone to pollution as evident from polluted water invasion from the canal.

2-D ERT models for Traverses 5 (Figure 4g) and 7 (Figure 4h) were taken across each other and away from the lagoon, to further see the effect of the incursion inland. Low resistivity (below 10 Ωm) observed at the near surface from both traverses is a clear indication of change in lithology and fluid content (the site is water-logged, not sand-fill and host brackish water evidently seen on the surface). This portion has been highly impacted by the accumulation of conductive waste water (brackish water) around the area and possibly saltwater from the lagoon may have polluted the groundwater within the first aquifer along the traverse as depicted by very low resistivity value toward the second part of Traverse 5 which corresponds with the first part of Traverse 7 (Figure 4h – the intercept referred). The second geoelectric layer is sand with resistivity of 106–500 Ωm, which is possibly represents the aquifer unit.

**Figure 6:**
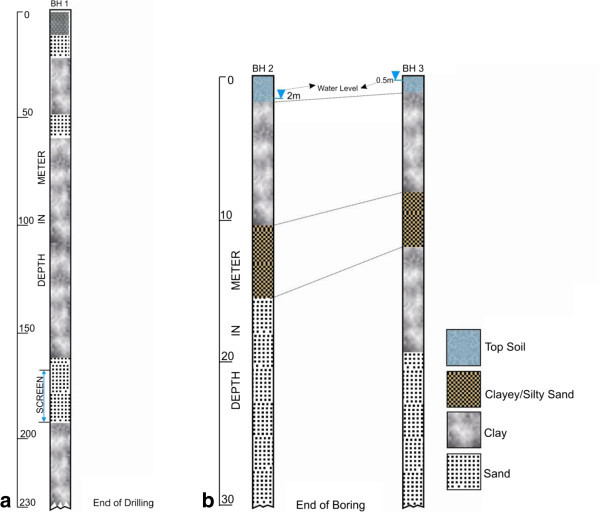
**Borehole log to which nearby ERT lines were correlated, a) borehole drilled in 1994 b) Shell Auger drill at the time of this survey.**

Depth range of 63–141 m was delineated from Traverse 6 (Figure 5a) established beside borehole B-1 (Figure 6a). Isolated near surface high resistivity within the sandy clay topsoil was reflective of buried utilities such as drainage and water, cables and canal. Because the traverse was located close to the water work station of the university, all pipes conducting water to the water station were buried in the topsoil around the traverse. Juxtaposing with borehole data the second sandy layer in the borehole was probably not mapped in the geoelectric layer because of the presence of saline water in the diminutive sandy layer which increases its conductivity and thus makes it impossible to be differentiated from underlying clayey layer. A long stretch of clay layer was mapped under this traverse, as observed from the log data. The 2-D ERT section (Figure 5b) along Traverse 8 depicts possible pollution from brackish water of the swamp.

Traverse 11 (Figure 5c) taken opposite Traverse 12, almost parallel but not across the stream dipping slightly towards the stream also reveals very low resistivity value(3.5–30 Ωm) towards the stream (third geoelectric layer) suggesting the possibility of infiltration of saline water from the stream at depth of about 18–70 m (surface positions of 170–250 m). This was mapped by both Schlumberger, double-dipole and pole-dipole methods. 2-D ERT model structure on Traverse 12 (Figure 5d) indicates low resistivity distributions towards the central portion of the traverse as a combination of water resistivity within the stream and its sediment as electrodes within these regions fell within the stream. This low resistivity structure is a reflection of the conductive nature of the stream (brackish water) and its sediment. The extension of this low resistivity structure outside the stream bank westward is suggestive of the possibility that the shallow aquifer in this area may have been polluted by the brackish water percolation through the porous topsoil. This stream could possibly serve as recharge unit for the aquifer.

Traverses 13 located close to the creek (Figure 5e), near the two Shell auger boreholes was marshy frequently flooded by the creek especially during raining season. Region close to the Lagoon exhibits very low resistivity structure reflective of saltwater intrusion from the creek as observed at surface distance of 210–284 m below 10 m depth. The affected layer was thought to be the first aquifer; its incursion by the lagoon water is an indication of progressive seawater intrusion to the inland aquifer within the campus. The Shell Auger borehole (Figure 6b) corroborated the 2-D ERT result for this area.

Traverse 14 located on dumpsite within the campus shows leachate impacted soil from the decomposing refuse materials characterized with resistivity below 10 Ωm. Pollution from dumpsite leacheate was mapped in the 2-D ERT inverted resistivity structure (Figure 5e). Traverse 15 was run along the road to the High Rise (staff quarters) parallel to the lagoon. The 2-D ERT section (Figure 5e) is reflective of subsurface conductivity. An interesting features (Figure 5e) reflective of buried utilities such as drainage, canal, buried water pipes, etc., that empty their content into the lagoon were clearly marked along this traverse and exhibits isolated near surface high resistivity structures within the top soil. The second geoelectric layer has been impacted by saltwater from the lagoon to a depth of about 28 m, though largely masked by the buried utilities.

### Geochemical analysis

**Figure 7:**
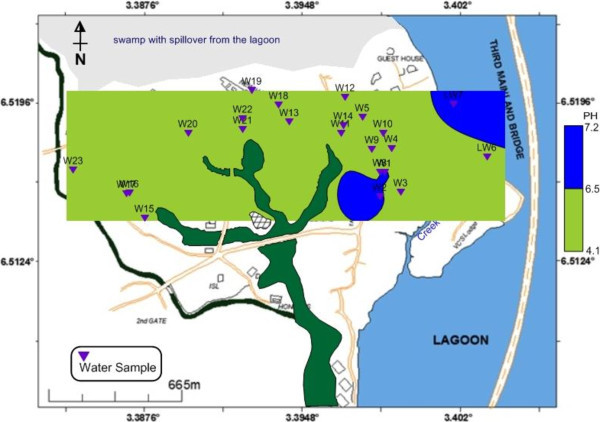
**pH distribution of borehole and lagoon water.**

**Figure 8:**
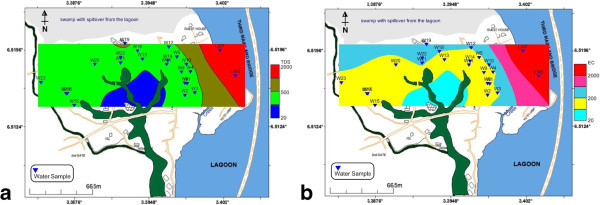
**Flow model of a) TDS and b) EC movement from the lagoon into coastal aquifers.(b)** depicts the provenance of coastal aquifer EC as the lagoon water.

The observed pH values between 4.21 and 7.08 put the groundwater in the acidic domain. Figure 7 shows that over 70% (red colour) of the water samples are acidic which is out of USEPA (2008) and USEPA (2009) standards. TDS values below 1 g/L are associated with fresh groundwater (Postma et al. 2007; Larsen et al. 2008; Jessen et al. 2008; Winkel et al. 2011) and these values correspond to an electrical resistivity below 100 mS/m (ρ_w_ > 10 ohm-m) (Tran et al., 2012). Using a TDS below 1 g/L (1000 mg/L) to separate fresh groundwater from saline groundwater, the groundwater within University of Lagos could be classified as fresh water as depicted in Figure 8a.

**Figure 9:**
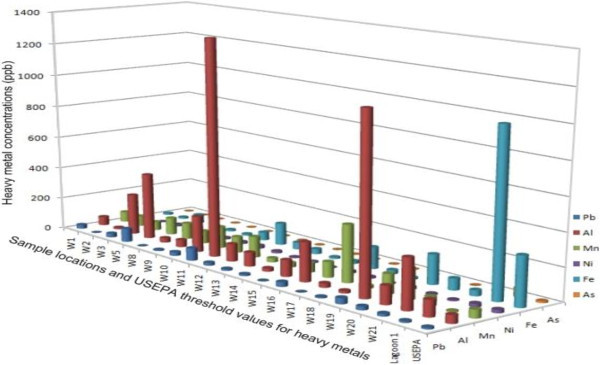
**Heavy metal distribution in the water samples and USEPA threshold value for each metal.**

Major ions analyzed are given in Table 1. The cations detected in high concentrations are Ca, Mg, P, S, K and silica but were not above the threshold values for drinking water. Significant among the macroelements is Na in which 14 samples exceeded the USEPA non-regulatory drinking-water advisory threshold. Trace elements of significant quantity in the water were extracted from over seventy elements analyzed for and the corresponding statistics shown in Table 2. Al, Fe, Pb, Mn, Br and Ni exceeded recommended standards in 60.87%, 4.35%, 65.2%, 18.78%, 100% and 13.04% of the total samples respectively (Figure 9). All the heavy metals are present in lagoon water at a very high concentration. Though detail chemistry and spatial evolution of the water samples will be presented in another paper currently been prepared, we will used the inflow of geochemical elements as evidence of incursion of saline/brackish water into the aquifers under the study area.

**Table 1:** **pH, TDS, EC and macroelements contents (mg/L) of water samples obtained from boreholes and lagoon in the area**

Physical parameter/major elements	Range	Mean	Standard deviation	Number of samples exceeding standards	Lagoon water	USEPA standards (2002,2009) (a, b, c, d)
pH	4.27-7.08	6.58	4.6	19	7.08	6.5-8.5^a,c^
TDS	18-312	88.9	58.4	0	2000*	500^a,c^
EC	37-630	178.7	117.8	0	3999	
Ca	2.76-23.19	9.2	5.9	0	249	
Mg	1.92-7.49	7.4	6.5	0	776	
Na	19.42-164.68	56.8	39.4	0	6511	30^b^-60^c^
K	o.66-11.03	26.05	64.39	0	223	
P	<0.02-0.16	0.059	0.041	0	2	
S	1.0-34	9.44	7.96	0	596	
Si	4.5-36.52	11.8	7.03	0	40	
Cl	14-286	79	70.3	1	8976	250^b,c^

a – USEPA maximum contaminant Level; b –USEPA Secondary Maximum Contaminant Level; c – National Primary Drinking Water Standard; * maximum recordable value of the instrument.

**Table 2:** **Summary of trace elements and heavy metals contents (μg/l) of water samples obtained from wells and boreholes around the dumpsite**

Trace element/heavy metal	Range (ppb)	Mean	Standard deviation	Lagoon water (ppb)	USEPA standard (2002, 2009) (a, b, c, d)
Br	7-951	304.68	230.68	32866	
Pb	0.1-76.8	24.93	23.65	39	10^d^
Al	11-1331	251.95	347.28	306	50^c^
Mn	24.41-348.37	96.56	70.97	146	^b^50-300^b^
Ni	2.4-30.1	11.38	7.6	34	20^d^
Fe	<10-463	117.14	110.18	1000	300^c,d^
As	<0.5-1.1	0.68	0.19	50	10^a,d^

a – USEPA maximum contaminant Level; b – NYSDOH Maximum Contaminant Level; c – USEPA Secondary Maximum Contaminant Level; d – National Primary Drinking Water Standard.

### Borehole data

One is the ways to constraint geophysical results is the use of a more reliable or proven geophysical method (for example Ayolabi et al., 2010) or by using information from other sources Sudha et al., (2009). The best approach still remains information from log data obtained from drilled holes since all other geophysical methods are indirect methods that depend on the inferences from signals received at the surface. Correlation inferred from this could be veritable tools to interpret other data.

2-D ERT line 6 run across a borehole drilled and logged in 1994. Borehole log shows 10 m thick sandy clay top soil followed by 11 m thick reddish sand, which is underlain by 28 m thick clay as shown in Figure 6a. A thin sandy layer of 11 m thick underlain the clay layer in alternation followed by a very thick layer of clay (100 m thick). Juxtaposing this with resistivity depth; there was no distinction between sandy clay topsoil and the underlying sand as both reflect resistivity variation interpreted as sand. The information that is not known is that between the time the borehole was drilled in 1994 and now if the present ground level is the same with what obtainable then. This would definitely affect the correlation of geoelectric layer obtained from our study and the geologic layer of the then subsurface. Nonetheless, the borehole would be used to correlate profiles around it (central part of the campus). Two other borehole sources used are Shell Auger boreholes (Figure 6b) drilled at the back of Faculty of Science (Traverse 13) a few days after the acquisition of 2-D ERT data.

## Discussion of results

### Saltwater incursion from the ERT data

The ERT resistivity-depth models show that some of the aquifer units were found to have been impacted by saline water due to incursion from the Lagoon. Generally, the polluted regions were characterized by very low resistivity value, less than 10 Ωm. Other sources of pollution observed from our results include brackish water infiltration from the creek, waste water accumulation and percolation through unlined drainage/canal within the university, waste water from adjoining canal, infiltration from polluted stream. Leachate from the dumpsite within the campus, which effect is still minimal now, may become another major source of pollution to the groundwater with time. We strongly perceived that daily excessive groundwater extraction by the over 20 functioning boreholes in the campus will aggravate the saline incursion with time. Taking Traverses 1, 2 and 9 (Figure 3) for example, with increasing pumping of groundwater from boreholes 4, 5, 8 and 22 (Figure 2), the aquifers under these boreholes become more prone to invasion of salty water in a transgressive flow.

### Groundwater quality

#### Physicochemical behaviours of the groundwater

Figure 8b plotted from the observed pH values depicts increasing electrical conductivity (EC) from mainland aquifers towards the lagoon, a situation that aptly suggests the influence of lagoon water salinity through gradual encroachment into the coastal aquifers as an evidence of the provenance of aquifer EC. The lagoon recharges the aquifers and since its water is saline, therefore the invaded aquifers become more conductive. Similar conductivity (resistivity) flow paths from the water body to the coastal aquifers were equally notice in the ERT survey (Traverses 1, 2, 3 and 9).

**Figure 10:**
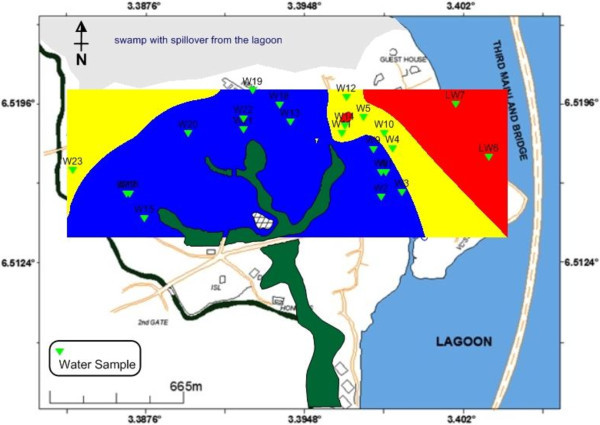
**Heavy metal provenance map.** The decreasing total contributions of heavy metals from the Lagoon towards inland are a possible evidence the Lagoon is encroaching into the coastal aquifer.

As the lagoon water transgresses and regresses (own to seasonal variations and specific gravity; liquid moving from region of high to lower concentrations), geochemical elements in the water also follow. This accounts for increasing pattern of the geochemical (and physical) elements from the mainland to the lagoon. Only Na macroelements exceeded the threshold values which apparently reflect in the electrical conductivity of the water. All the heavy metals are present in the lagoon water at very high concentrations and gradually infiltrate the nearby groundwater aquifers possibly during aquifer recharge or intrusion as depicted by increasing concentrations from the mainland toward the lagoon (Figure 10). Continuous flow of these metals toward the mainland could become a threat and source of enrichment of the aquifers with heavy metals that may not be environmentally benign.

### Pollution index (PI)

The pollution index was used in this study to evaluate the degree of trace metal contamination in water samples (Chon, *et al*., 1991; Kim *et al.,*^1998^; Emoyan *et al.,*^2005^; NIER 2007; Odukoya and Abimbola 2010). The tolerable level is the element concentration in the water considered safe for human consumption (Lee *et al*., 1998). The USEPA national drinking water standards (2009) were used as tolerable level for water and the pollution index (PI) was calculated by the equation:

Where Mc = Heavy metal concentration in water; T_L_ = Tolerable Level and N_m_ = Number of Heavy metals.

**Figure 11:**
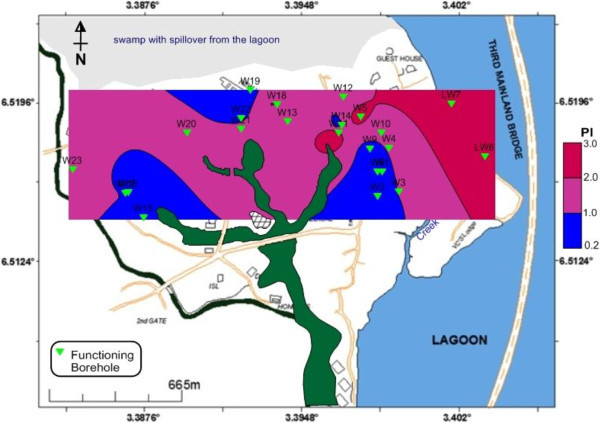
**Pollution index (PI) deduced from heavy metals.** Lagoon water as possible source of the pollution is indicated by increasing PI from coastline aquifer towards the lagoon.

**Figure 12:**
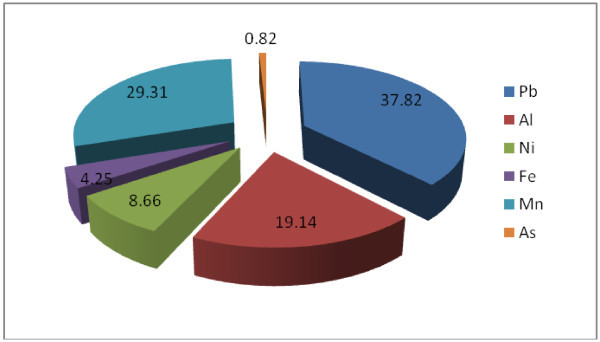
**Percentage contributed by significant heavy metals to PI.**

Water sample with Pollution Index (PI) greater than 1 is regarded as being contaminated. More than 50% of groundwater in the area is polluted having PI between 1.0 and 2.86. Most importantly, Figure 11 depicts the provenance of the PI as the lagoon with increasing contamination level from the coastal aquifers to the lagoon. Similar flow path has been earlier observed from EC, TDS, trace elements and the resistivity. Lead (Pb) contributed the highest percentage (37.8%) to the Pollution Index, followed closely by Mn, Al, Ni, Fe and As (Figure 12).

## Conclusion

The combined geophysical and physicochemical evidence obtained from the mapping exercise carried out in this study revealed that the coastal aquifer under the University of Lagos campus has been intruded by saltwater from the adjacent lagoon. The hydraulic gradient enables water flow from the lagoon into the aquifer. 2-D ERT data were acquired at fifteen locations to obtain information about subsurface lithology and fluid conductivity (in turn resistivity). This was coupled with physical and chemical analyses of twenty borehole water samples from functioning boreholes in the campus. A depth of 30–226 m beneath the surface was penetrated through the 2-D ERT imaging. Saline/brackish water intrusion was mapped out under traverses close to the lagoon and the carnal that encircle the campus, which has affected the first and the second aquifers. Five major sources of pollution have been identified from the 2-D ERT Survey – 1. Pollution from the Lagoon; 2. Pollution from the Creek; 3. Pollution from the adjoining canal; 4. Pollution from waste water drainage within the University; 5. Pollution from polluted stream that flows through the University to the Lagoon. Effort is needed to prevent the third and fourth aquifer from pollution as these may be the only sources of fresh water in the advent of drought with attendant exponential incursion of saline water from the lagoon due to mass withdrawal of fresh water upland. Waste water canal/drainage within the university community must be properly lined with concrete material to prevent further percolation of waste water into the aquifer.

Water samples analyzed for physical properties and chemical constituents, including inorganic major ions, nutrients and trace elements revealed that each borehole sampled had at least one constituent that exceeded a USEPA, 2009 drinking-water standard, Maximum Contaminant Levels (MCLs) or Secondary Maximum Contaminant Levels set by the U.S. Environmental Protection Agency (USEPA) for major and trace elements. We observed progressive decrease in TDS and EC from the lagoon to the inland aquifer as indication of underground flow of the lagoon water into the aquifers. Similar trend in heavy metal distribution was also noticed. It is concluded that the excessive groundwater extraction is responsible for saline intrusion into the coastline aquifer, since as much as 70% of the thirty water boreholes located on the campus were still functioning at the time of this survey.
